# The Structural, Photocatalytic Property Characterization and Enhanced Photocatalytic Activities of Novel Photocatalysts Bi_2_GaSbO_7_ and Bi_2_InSbO_7_ during Visible Light Irradiation

**DOI:** 10.3390/ma9100801

**Published:** 2016-09-27

**Authors:** Jingfei Luan, Yue Shen, Yanyan Li, Yaron Paz

**Affiliations:** 1 State Key Laboratory of Pollution Control and Resource Reuse, School of the Environment, Nanjing University, Nanjing 210093, China; yueshen_sally@outlook.com (Y.S.); yanyanli_2016@sina.com (Y.L.); 2 Department of Chemical Engineering, Technion-Israel Institute of Technology, Haifa 32000, Israel; paz@tx.technion.ac.il

**Keywords:** photocatalysts, Bi_2_GaSbO_7_, Bi_2_InSbO_7_, methylene blue, photocatalytic degradation, visible light irradiation

## Abstract

In order to develop original and efficient visible light response photocatalysts for degrading organic pollutants in wastewater, new photocatalysts Bi_2_GaSbO_7_ and Bi_2_InSbO_7_ were firstly synthesized by a solid-state reaction method and their chemical, physical and structural properties were characterized. Bi_2_GaSbO_7_ and Bi_2_InSbO_7_ were crystallized with a pyrochlore-type structure and the lattice parameter of Bi_2_GaSbO_7_ or Bi_2_InSbO_7_ was 10.356497 Å or 10.666031 Å. The band gap of Bi_2_GaSbO_7_ or Bi_2_InSbO_7_ was estimated to be 2.59 eV or 2.54 eV. Compared with nitrogen doped TiO_2_, Bi_2_GaSbO_7_ and Bi_2_InSbO_7_, both showed excellent photocatalytic activities for degrading methylene blue during visible light irradiation due to their narrower band gaps and higher crystallization perfection. Bi_2_GaSbO_7_ showed higher catalytic activity compared with Bi_2_InSbO_7_. The photocatalytic degradation of methylene blue followed by the first-order reaction kinetics and the first-order rate constant was 0.01470 min^−1^, 0.00967 min^−1^ or 0.00259 min^−1^ with Bi_2_GaSbO_7_, Bi_2_InSbO_7_ or nitrogen doped TiO_2_ as a catalyst. The evolution of CO_2_ and the removal of total organic carbon were successfully measured and these results indicated continuous mineralization of methylene blue during the photocatalytic process. The possible degradation scheme and pathway of methylene blue was also analyzed. Bi_2_GaSbO_7_ and Bi_2_InSbO_7_ photocatalysts both had great potential to purify textile industry wastewater.

## 1. Introduction

Dye contaminants from textile wastewater were difficult to treat for their high chroma, high chemical oxygen demand content and complicated ingredients. Some conventional methods including biodegradation [1,2,3], electrochemistry [3,4,5,6], adsorption [7,8,9], and flocculation−precipitation [10,11] had been exploited to degrade those dye contaminates, but there still existed a serious of problems with them. Methylene blue (MB), usually adopted as dyestuff, was one of the most common dye contaminants.

Photocatalysis had gained great development since photocatalytic reaction was found in 1972 [12]. Photocatalytic degradation of the pollutants in wastewater entailed a chain of advantages including conserving energy and little secondary pollution; it had therefore gradually attracted more and more attention in textile wastewater treatment. Metal oxides [13,14,15,16,17,18,19,20,21,22,23,24] and metal sulfides [21,22,23,24,25,26,27,28,29,30,31,32,33] were the most common semiconductor photocatalysts. Among metal oxides, anatase TiO_2_ was investigated most repeatedly owing to its non-toxic property, excellent stability and low cost. However, with a wider band gap (3.2 eV), anatase TiO_2_ only efficiently absorbed ultraviolet light which occupied only 5% of the solar energy, and thus failed to make good use of optical energy. In order to make the best use of visible light which occupied 43% of sunlight, developing visible light responsive photocatalysts was an inevitable tendency in the field of photocatalysis research, which could be embodied from abundant endeavors of previous scholars in realizing the degradation of the pollutants during visible light irradiation by the method of iron doping [34,35,36], forming heterojunction [37,38,39,40,41,42] or photosensitization [43,44,45,46,47,48]. Several years ago, Zou and Arakawa [49,50] found that two types of metal oxides, ABO_4_ and A_2_B_2_O_7_, had great potential for photocatalytic H_2_ production during visible light irradiation. It was well known that minute changes in internal structure of the semiconductor photocatalysts would presumably promote the separation of photogenerated electrons and holes and thus improve photocatalytic activities. Zou et al. synthetized Bi_2_MNbO_7_ (M = Al, Ga, In, Y or Fe) [51,52,53] which was one remarkable representative of the family of A_2_B_2_O_7_ compounds with the A^3+^_2_B^4+^_2_O_7_ pyrochlore structure by substituting B^4+^ sites in A^3+^_2_B^4+^_2_O_7_ for M^3+^ (M^3+^ = Al^3+^, Ga^3+^, In^3+^) and Nb^5+^. Similarly, previous studies had reported Bi_2_GaVO_7_ [54] and Bi_2_SbVO_7_ [55] by element doping, which had realized visible-light photocatalytic degradation and H_2_ production. Previous works indicated that the Ga^3+^ and In^3+^ ions could influence the band gap and the electronic structure of the compound photocatalysts, which was expected to cause the different photocatalytic activity [56,57].

As an important element with higher electron drift velocity and mobility, antimony (Sb) has been extensively studied as a good dopant candidate for enhancing the electron transfer rate of semiconductors [58]. Omidi et al. [59] evaluated the photocatalytic activity of Sb-doped ZnO nanostructures (0 ≤ mol fraction of Sb^3+^ ions ≤ 0.15) for the photodegradation of MB. In addition, the acquired results showed that doping the ZnO nanostructures with 0.03 mol fraction of Sb^3+^ ions increased the reaction rate by about three times, indicating that the decreasing recombination of charge carriers could enhance the photocatalytic activity. Al-Hamdi et al. [60] reported that Sb-doped dioxide (SnO_2_) nanoparticles with different Sb concentrations (at % = 0, 2, 4 and 6), which was prepared by a sol–gel method, could degrade 12%, 45%, 71% and 97% of phenol in the mineralization process under UV irradiation for 120 min, which showed higher photocatalytic activity than the undoped SnO_2_ catalyst. These previous reports have shown that moderate Sb doped on the photocatalysts could greatly enhance the photocatalytic activity.

In this paper, new photocatalysts, Bi_2_GaSbO_7_ and Bi_2_InSbO_7_, were synthetized by doping element Ga or In with a solid-state reaction method. Meanwhile, the structural properties of Bi_2_GaSbO_7_ and Bi_2_InSbO_7_ were also characterized and their photocatalytic activities were also examined in degrading MB solution compared with N-doped TiO_2_, which had achieved the visible light response.

## 2. Materials and Methods

### 2.1. Synthesis of Bi_2_GaSbO_7_, Bi_2_InSbO_7_ and N-doped TiO_2_ Photocatalysts

New Bi_2_GaSbO_7_ and Bi_2_InSbO_7_ samples were firstly synthesized by a solid-state reaction method. Firstly, for the sake of the synthesis of Bi_2_GaSbO_7_, Bi_2_O_3_, Ga_2_O_3_ and Sb_2_O_5_ with a purity of 99.99% (Sinopharm Group Chemical Reagent Co., Ltd., Shanghai, China) were obtained by an atomic ratio of 2:1:1 to serve as raw materials. All powders were dried at 200 °C for 4 h before synthesis. In order to synthesize Bi_2_GaSbO_7_, the precursors were fully mingled with each other, then pressed into small columns and put into an alumina crucible (Shenyang Crucible Co., Ltd., Shenyang, China). Eventually, calcination was performed at 1100 °C for 40 h in an electric furnace (KSL 1700X, Hefei Kejing Materials Technology Co., Ltd., Hefei, China). Accordingly, Bi_2_O_3_, In_2_O_3_ and Sb_2_O_5_ with a purity of 99.99% (Sinopharm Group Chemical Reagent Co., Ltd., Shanghai, China) were obtained by an atomic ratio of 2:1:1 for the preparation of Bi_2_InSbO_7_. The synthesization procedure of Bi_2_InSbO_7_ was similar to that of Bi_2_GaSbO_7_, just the calcination was performed at 1070 °C for 30 h during mixed powder in the alumina crucible. The preparation of N-doped TiO_2_ was by the sol–gel method which was mentioned in our previous studies [61].

### 2.2. Characterization

In our paper, we adopted the X-ray diffraction method (XRD, D/MAX-RB, Rigaku Corporation, Tokyo, Japan) with Cu Ka radiation (*λ* = 1.54056 angstrom) to confirm the crystal structures of Bi_2_GaSbO_7_ and Bi_2_InSbO_7_. The patterns of Bi_2_GaSbO_7_ and Bi_2_InSbO_7_ were recorded at 295 K with a step–scan procedure in the range of 2*θ* = 10°–100° (for Bi_2_GaSbO_7_) or 10°–95° (for Bi_2_InSbO_7_). The step interval was 0.02° and the time per step was 1 s. The transmission electron microscopy (TEM, Tecnal F20 S-Twin, FEI Corporation, Hillsboro, OR, USA) was used to observe the surface state and structure of the photocatalysts. The Malvern’s mastersize-2000 particle size analyzer (Malvern Instruments Ltd., Malvern, UK) was utilized to measure the particle size of the photocatalysts. We also utilized X-ray photoelectron spectroscopy (XPS, ESCALABMK-2, VG Scientific Ltd., London, UK) to determine the Bi^3+^ content, Ga^3+^ content, Sb^5+^ content, In^3+^ content and O^2−^ content of Bi_2_GaSbO_7_ and Bi_2_InSbO_7_. The chemical composition of Bi_2_GaSbO_7_ and Bi_2_InSbO_7_ was determined by scanning the electron microscope-X-ray energy dispersion spectrum (SEM–EDS, LEO 1530VP, LEO Corporation, Dresden, Germany). The surface areas of Bi_2_GaSbO_7_ and Bi_2_InSbO_7_ were measured by the Brunauere–Emmette–Teller (BET) method (MS-21, Quantachrome Instruments Corporation, Boynton Beach, FL, USA) with N_2_ adsorption at liquid nitrogen temperature. Their diffuse reflectance spectrums were analyzed by a UV-visible spectrophotometer (Shimadzu UV-2550 UV-Visible spectrometer, Kyoto, Japan).

### 2.3. Photocatalytic Properties Test

MB (C_16_H_18_ClN_3_S) (Tianjin Bodi Chemical Co., Ltd., Tianjin, China) served as our objective pollutant. The whole photocatalytic activity process was as follows: firstly, we prepared 300 mL MB aqueous solution in quartz tubes whose initial concentration was 0.025 mmol·L^−1^ and initial PH value was 7.0. Then, 0.8 g photocatalyst powder of N-doped TiO_2_, Bi_2_GaSbO_7_ or Bi_2_InSbO_7_ was placed into every quartz tube, respectively. In order to ensure the establishment of an adsorption/desorption equilibrium among photocatalysts, the MB dye and atmospheric oxygen, above per solution was magnetically stirred in the dark for 45 min. In our paper, we employed a 500 W Xenon lamp (*λ* > 420 nm), which utilized a 420 nm cutoff filter as a visible-light source. The photoreaction was carried out in a photochemical reaction apparatus (Nanjing Xujiang Machine Plant, Nanjing, China). During visible light illumination, the MB dye pollution was stirred by a magnetic stirrer and the photocatalyst powder was kept suspended in the solution. The filtrate was subsequently measured by a Shimadzu UV-2450 UV-visible spectrometer (Kyoto, Japan) with the detecting wavelength at 665 nm. The identification of MB and the degradation intermediate products of MB were measured by a liquid chromatograph-mass spectrometer (LC–MS, Thermo Quest LCQ Duo, Silicon Valley, CA, USA, Beta Basic-C_18_ HPLC column: 150 × 2.1 mm^2^, ID of 5 μm, Finnigan, Thermo, Silicon Valley, CA, USA). Here, post-photocatalysis solution (20 μL) was injected automatically into the LC–MS system. The eluent contained 60% methanol and 40% water, and the flow rate was 0.2 mL·min^−1^. MS conditions included an electrospray ionization interface, a capillary temperature of 27 °C with a voltage of 19.00 V, a spray voltage of 5000 V and a constant sheath gas flow rate. The spectrum was acquired in the negative ion scan mode, sweeping the *m*/*z* range from 50 to 600. Evolution of CO_2_ was analyzed with an intersmat™ IGC120-MB gas chromatograph (6890 N, Agilent Technologies, Palo Alto, CA, USA) equipped with a porapack Q column (3 m in length and with an inner diameter of 0.25 in.), which was connected to a catharometer detector. The total organic carbon (TOC) concentration was determined with a TOC analyzer (TOC-5000, Shimadzu Corporation, Kyoto, Japan). The photonic efficiency was calculated according to the following equation [62,63]:

(1) $$\xi =R/I_{0}$$

where *ξ* was the photonic efficiency (%), and *R* was the rate of MB degradation (mol·L^−1^·s^−1^), and *I*_0_ was the incident photon flux (Einstein·L^−1^·s^−1^). The incident photon flux *I*_0_ which was measured by a radiometer (Model FZ-A, Photoelectric Instrument Factory Beijing Normal University, Beijing, China) was determined to be 4.76 × 10^6^ Einstein·L^−1^·s^−1^ under visible light irradiation (wavelength range of 400–700 nm).

## 3. Results and Discussion

### 3.1. Characterization

**Figure 1:**
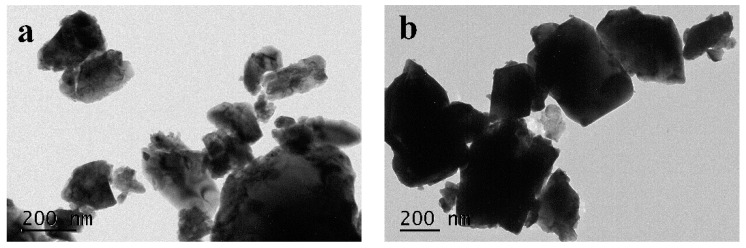
TEM images of (**a**) Bi_2_GaSbO_7_ and (**b**) Bi_2_InSbO_7_ with high magnification.

**Figure 2:**
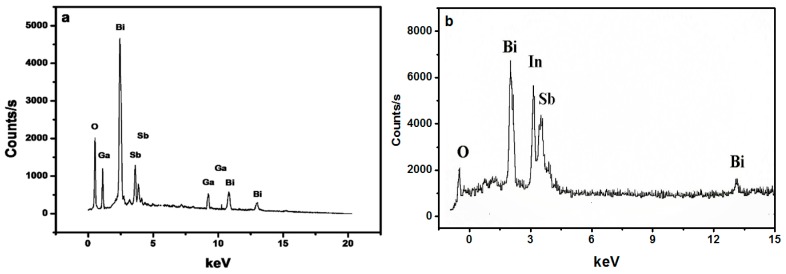
SEM–EDS spectra taken from (**a**) Bi_2_GaSbO_7_ and (**b**) Bi_2_InSbO_7_.

Figure 1a,b shows the TEM images of Bi_2_GaSbO_7_ and Bi_2_InSbO_7_ with high magnification. We could observe from the images of Bi_2_GaSbO_7_ and Bi_2_InSbO_7_ that their particles presented a similar oblate spheroid appearance and that their distribution was relatively uniform. The average particle size of Bi_2_GaSbO_7_ approached 190 nm, which was smaller than that of Bi_2_InSbO_7_, whose average particle size approached 390 nm. We could observe from the BET results that the specific surface area of Bi_2_GaSbO_7_ approached 2.36 m^2^/g, which was bigger than that of Bi_2_InSbO_7_, whose specific surface area approached 1.82 m^2^/g. It was clear that the BET results were consistent with the TEM results, indicating that the samples with small average particle size would have a higher specific surface area. Figure 2a,b shows the SEM–EDS spectra taken from Bi_2_GaSbO_7_ and Bi_2_InSbO_7_. It could be seen from Figure 2a,b that the superfluous peaks did not exist in the spectra of Bi_2_GaSbO_7_ and Bi_2_InSbO_7_, meaning that Bi_2_GaSbO_7_ and Bi_2_InSbO_7_ crystals were both pure phase without impure elements.

In this paper, X-ray photoelectron spectroscopy analysis techniques were utilized to reveal the surface chemical compositions and the valence states of various elements in Bi_2_GaSbO_7_ and Bi_2_InSbO_7_. The various elemental peaks which are corresponding to specific binding energies are given in Table 1. Analysis results of the full XPS spectra were as follows: the prepared Bi_2_GaSbO_7_ sample contained Bi, Ga, Sb and O elements. Similarly, the prepared Bi_2_InSbO_7_ sample contained Bi, In, Sb and O elements. These results also uncovered that Bi_2_GaSbO_7_ crystal or Bi_2_InSbO_7_ crystal were both at a high pure phase. Moreover, the analysis results of the XPS spectra also manifested that the valence of Bi, Ga, Sb, In or O from Bi_2_GaSbO_7_ and Bi_2_InSbO_7_ was +3, +3, +5, +3 or −2, respectively. Eventually, according to our comprehensive XPS and SEM–EDS analyses, as for Bi_2_GaSbO_7_, the mean atomic ratio of Bi, Ga, Sb and O was 2.00:0.98:1.02:6.98. As for Bi_2_InSbO_7_, the mean atomic ratio of Bi, In, Sb and O was 2.00:0.99:1.01:6.99.

**Figure 3:**
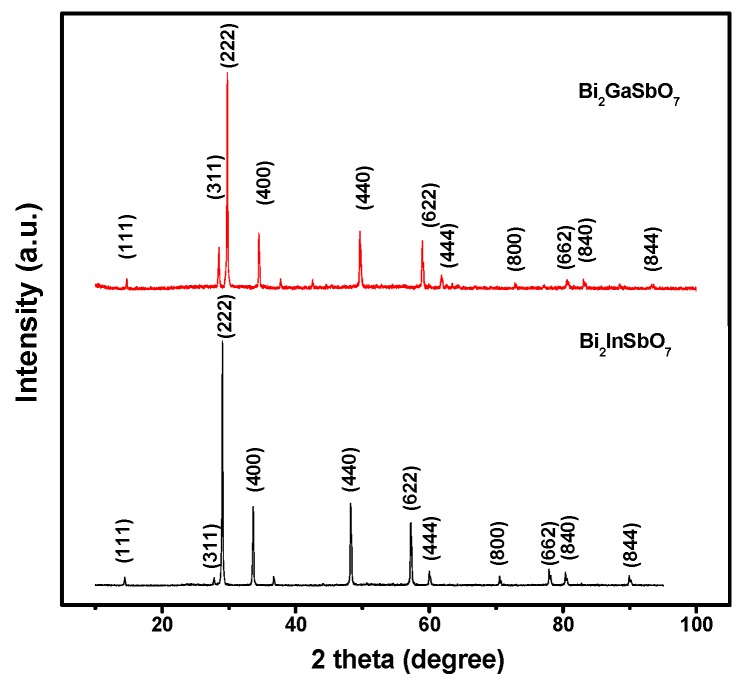
X-ray powder diffraction patterns of Bi_2_GaSbO_7_ and Bi_2_InSbO_7_.

**Figure 4:**
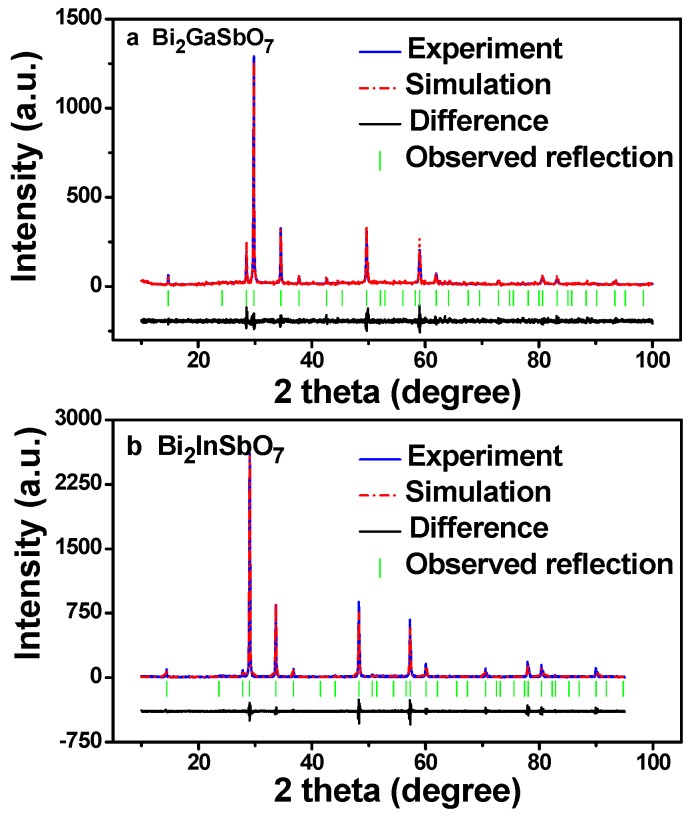
The Pawley refinement results of XRD data for (**a**) Bi_2_GaSbO_7_ and (**b**) Bi_2_InSbO_7_.

Figure 3 presents the X-ray powder diffraction patterns of Bi_2_GaSbO_7_ and Bi_2_InSbO_7_, respectively. We could judge from Figure 3 that Bi_2_GaSbO_7_ crystal or Bi_2_InSbO_7_ crystal was single phase. Figure 4a,b shows the Pawley refinement results of XRD data for Bi_2_GaSbO_7_ and Bi_2_InSbO_7_. The refined outcomes from Figure 4a,b displayed that the actual intensities of Bi_2_GaSbO_7_ or Bi_2_InSbO_7_ were both highly in accordance with the intensities of the pyrochlore-type structure with a cubic crystal system and a space group *Fd3m* (O atoms were included in the model), indicating that Bi_2_GaSbO_7_ and Bi_2_InSbO_7_ indeed formed the same crystal structure. The atomic coordinates and structural parameters of Bi_2_GaSbO_7_ and Bi_2_InSbO_7_ are listed in Table 2 and Table 3, respectively. Above results showed that the lattice parameter *a* of Bi_2_GaSbO_7_ was 10.356497 Å, which was slightly lower than that of Bi_2_InSbO_7_ whose lattice parameter *a* was 10.666031 Å. From the SEM–EDS spectra and XPS spectra which were taken from Bi_2_GaSbO_7_ and Bi_2_InSbO_7_, we had known that Bi_2_GaSbO_7_ crystal or Bi_2_InSbO_7_ crystal was both pure phase. Therefore, excluding the effects of impurities, we could deduce that the difference between the lattice parameter *a* for Bi_2_GaSbO_7_ and Bi_2_InSbO_7_ was perhaps concerned with M ionic radii which belonged to Bi_2_MSbO_7_. The reason was that the ionic radii of Ga^3+^ (0.62 Å) was minutely lower than that of In^3+^ (0.92 Å). Lastly, all the diffraction peaks (222), (400), (440), (622), (444), (800), (662), (840), (844) for Bi_2_GaSbO_7_ and Bi_2_InSbO_7_ were successfully indexed according to the lattice constant and above space group.

**Figure 5:**
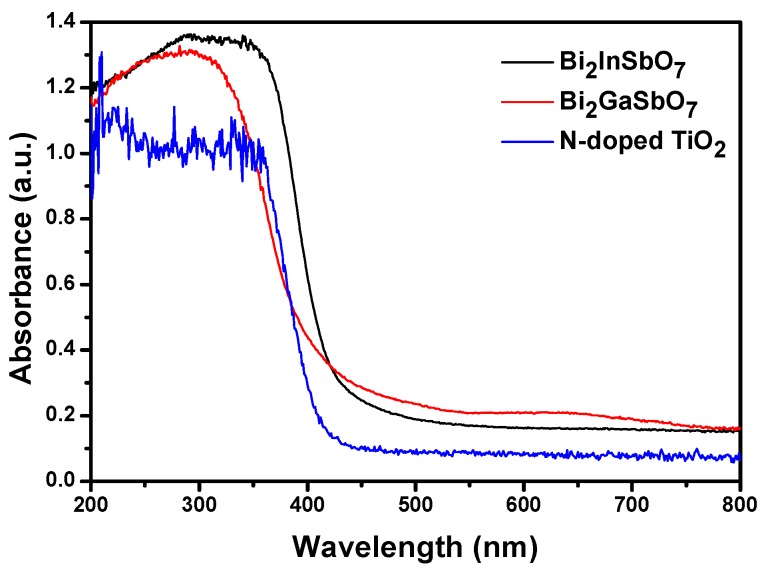
Diffuse reflection spectra of Bi_2_GaSbO_7_, Bi_2_InSbO_7_ and N-doped TiO_2_.

Figure 5 presents the diffuse reflection spectra of Bi_2_GaSbO_7_ and Bi_2_InSbO_7_, respectively. Compared with N-doped TiO_2_ whose absorption edge was about 445 nm, the absorption spectrum of newly prepared photocatalyst Bi_2_GaSbO_7_ or Bi_2_InSbO_7_ was estimated to be 480 nm or 490 nm, respectively, implicating that they had sizable potential to realize visible light response. The maximum absorption wavelength of MB was detected by an ultraviolet spectrophotometer, while the diffuse reflection spectra of Bi_2_GaSbO_7_, Bi_2_InSbO_7_ or N-doped TiO_2_ was detected by ultraviolet spectrophotometer with integrating sphere. In addition, the above two testing methods were totally different. Furthermore, the absorbance was obtained from the reflectance data and scattering should also be taken into consideration in data conversion from reflectance into absorbance, which was the reason why the ordinate of the diffuse reflection spectra in Figure 5 was absorbance. 

We realized that absorbance could not be proportional to 1-transmission, thus the absorbance was calculated using the Kubelka–Munk transformation method in our experiment. For a crystalline semiconductor compound, the optical absorption near the band edge followed the equation [64,65]: 

*αhν* = A × (*hν* − *E*_g_)*^n^* (2)

**Figure 6:**
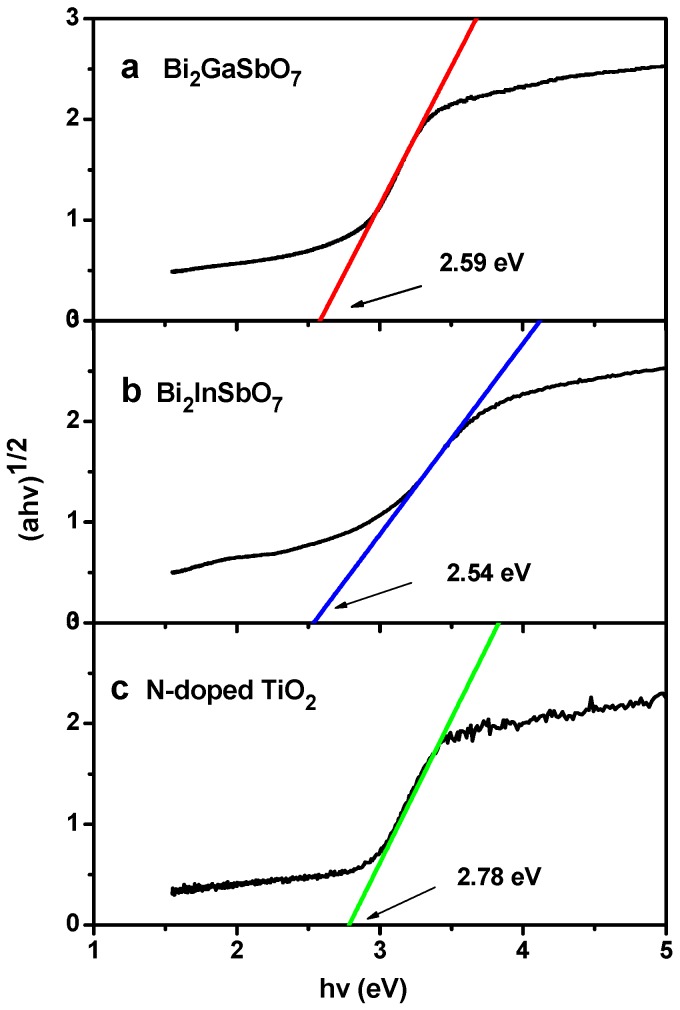
Plot of (*αhν*)^1/2^ versus *hν* for (**a**) Bi_2_GaSbO_7_; (**b**) Bi_2_InSbO_7_ and (**c**) N-doped TiO_2_.

Here, A, *α*, *E*_g_ and *ν* denoted proportional constant, absorption coefficient, band gap and light frequency, respectively. In this equation, *n* determined the character of the transition in a semiconductor compound. *E*_g_ and *n* could be calculated by the following steps: (i) plotting ln(*αhν*) versus ln(*hν* − *E*_g_) assuming an approximate value of *E*_g_; (ii) deducing the value of *n* according to the slope in this graph; (iii) refining the value of *E*_g_ by plotting (*αhν*)^1/*n*^ versus *hν* and extrapolating the plot to (*αhν*)^1/*n*^ = 0. According to this method, we first estimated that the value of *n* for Bi_2_GaSbO_7_ or Bi_2_InSbO_7_ was 2, indicating that the optical transition for Bi_2_GaSbO_7_ or Bi_2_InSbO_7_ is indirectly allowed. Figure 6 presents the plot of (*αhν*)^1/*n*^ versus *hν* for Bi_2_GaSbO_7_ and Bi_2_InSbO_7_. It could be found that the value of *E*_g_ for Bi_2_GaSbO_7_, Bi_2_InSbO_7_ or N-doped TiO_2_ was calculated to be 2.59 eV, 2.54 eV or 2.78 eV.

### 3.2. Photocatalytic Properties of Bi_2_GaSbO_7_ and Bi_2_InSbO_7_ Photocatalysts

**Figure 7:**
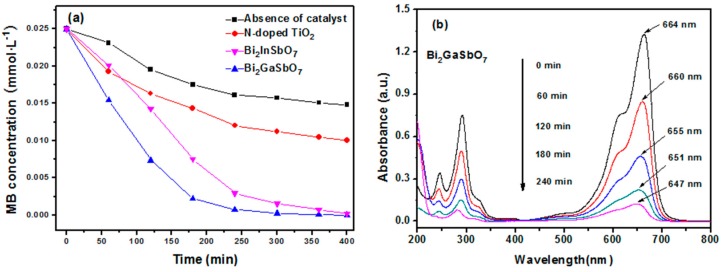
(**a**) Photocatalytic degradation of methylene blue under visible light irradiation in the presence of Bi_2_GaSbO_7_, Bi_2_InSbO_7_, N-doped TiO_2_ as well as in the absence of a photocatalyst; (**b**) Temporal UV-vis absorption spectral changes during the photocatalytic degradation of MB (0.025 mmol/L, pH = 7) in aqueous Bi_2_GaSbO_7_ suspensions.

From the UV-vis spectra of Bi_2_GaSbO_7_ and Bi_2_InSbO_7_, we had analyzed that both of the novel photocatalysts sent a strong absorption signal in the visible light region. Therefore, we expected that they could have the potential to degrade organic pollutants under visible light irradiation. In order to evaluate their visible light photocatalytic degradation capabilities, we listed N-doped TiO_2_ as a referential photocatalyst. Figure 7a presents the kinetics of MB degradation with Bi_2_GaSbO_7_, Bi_2_InSbO_7_, N-doped TiO_2_ as well as in the absence of a photocatalyst under visible light irradiation (>420 nm). Consistent with our expectations, as time went by, the color of the MB solution gradually shallowed and the concentration of MB gradually declined in our measurements in the absence of a photocatalyst. After visible light irradiation for 400 min, the removal rate of MB was estimated to be 99.75%, 98.95%, 59.92% or 40.6% with Bi_2_GaSbO_7_, Bi_2_InSbO_7_, N-doped TiO_2_ as catalyst, as well as in the absence of a photocatalyst, respectively. The sharp decrease in the concentration of MB under visible light irradiation from 0 to 120 min was mainly due to the adsorption of MB on the surface of Bi_2_GaSbO_7_, Bi_2_InSbO_7_ or N-doped TiO_2_ as a photocatalyst [66]. In the meantime, the photocatalytic degradation of MB with Bi_2_GaSbO_7_, Bi_2_InSbO_7_ or N-doped TiO_2_ as a catalyst also played a significant role compared with the absence of a photocatalyst under visible light irradiation in this sharp decrease. In addition, the slower speed of MB degradation by using Bi_2_GaSbO_7_, Bi_2_InSbO_7_ or N-doped TiO_2_ as a photocatalyst during the later reaction process could be the result of as-prepared samples surface blocking by adsorbed MB degradation byproducts [67]. Moreover, the photocatalytic degradation rate of MB was 1.039 × 10^−9^ mol·L^−1^·s^−1^, 1.031 × 10^−9^ mol·L^−1^·s^−1^ or 0.624 × 10^−9^ mol·L^−1^·s^−1^ with Bi_2_GaSbO_7_, Bi_2_InSbO_7_ or N-doped TiO_2_ as a catalyst during 400 min of visible light irradiation, respectively. The self-degradation rate of MB was 0.422 × 10^−9^ mol·L^−1^·s^−1^ without a catalyst. Furthermore, the photonic efficiency was estimated to be 0.0218% (*λ* = 420 nm), 0.0217% (*λ* = 420 nm) or 0.0131% (*λ* = 420 nm) with Bi_2_GaSbO_7_, Bi_2_InSbO_7_ or N-doped TiO_2_ as a catalyst, indicating that the sufficient use of a large number of photons could lead to the production of a large number of electron/hole pairs which were responsible for the photocatalytic degradation reaction directly and/or indirectly [68]. According to above results, it was apparent that Bi_2_GaSbO_7_ and Bi_2_InSbO_7_ harvested the highest photocatalytic degradation rate and photonic efficiency compared with N-doped TiO_2_ for degrading MB. The decolored MB solution and the decrease of MB concentration reflected from Figure 7a might ascribe to the destruction of chromophore and the thorough degradation of the whole MB molecular [69]. We have verified our conjecture by detecting the mount variation of TOC and CO_2_ during MB degradation. 

Figure 7b presents the UV-vis spectral changes during the photodegradation of MB with Bi_2_GaSbO_7_ as a photocatalyst. Noticeably, we could observe a subtle blue shift in the maximum absorbance of MB in the spectral changes by using Bi_2_GaSbO_7_ as a photocatalyst under visible light irradiation, indicating the rather facile cleavage of the whole conjugated chromophore structure [70]. This blue shift in the maximum absorbance of MB also proved the existence of some photodegradation intermediate products of MB during the photocatalytic degradation of MB under visible light irradiation in the presence of Bi_2_GaSbO_7_.

**Figure 8:**
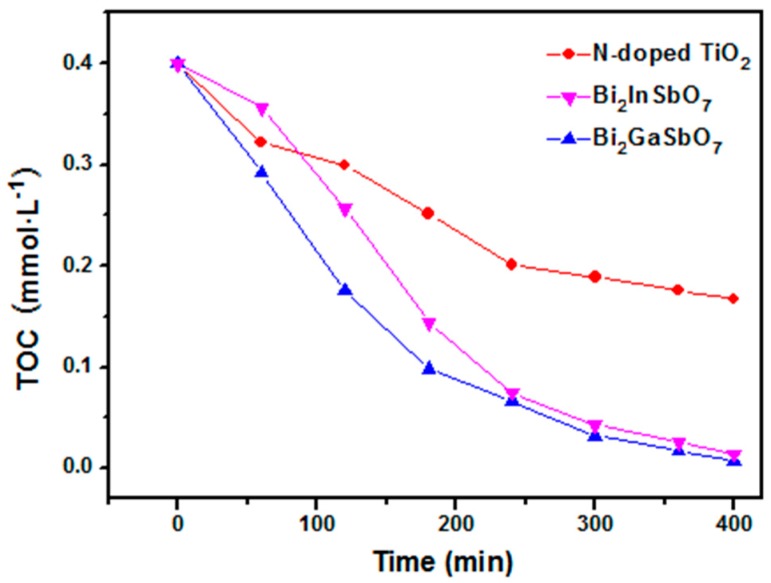
Disappearance of the total organic carbon (TOC) during the photocatalytic degradation of methylene blue with Bi_2_GaSbO_7_, Bi_2_InSbO_7_ or N-doped TiO_2_ as a catalyst under visible light irradiation.

Figure 8 shows the change of TOC for the photocatalytic degradation of MB during visible light irradiation with Bi_2_GaSbO_7_, Bi_2_InSbO_7_ or N-doped TiO_2_ as a photocatalyst, which is consistent with the tendency shown in Figure 7. The gradual decrease of TOC represented the gradual disappearance of organic carbon when the MB solution which contained Bi_2_GaSbO_7_, Bi_2_InSbO_7_ or N-doped TiO_2_ was exposed under visible light irradiation and the removal rate of TOC was 98.23%, 96.42% or 58.08% with Bi_2_GaSbO_7_, Bi_2_InSbO_7_ or N-doped TiO_2_ as a catalyst after visible light irradiation for 400 min. In addition, the reactions stopped when the light was turned off in this experiment, which showed the obvious light response, suggesting that MB had been converted to other kinds of byproducts and the organic carbon in the MB had not been decomposed to CO_2_ [71].

**Figure 9:**
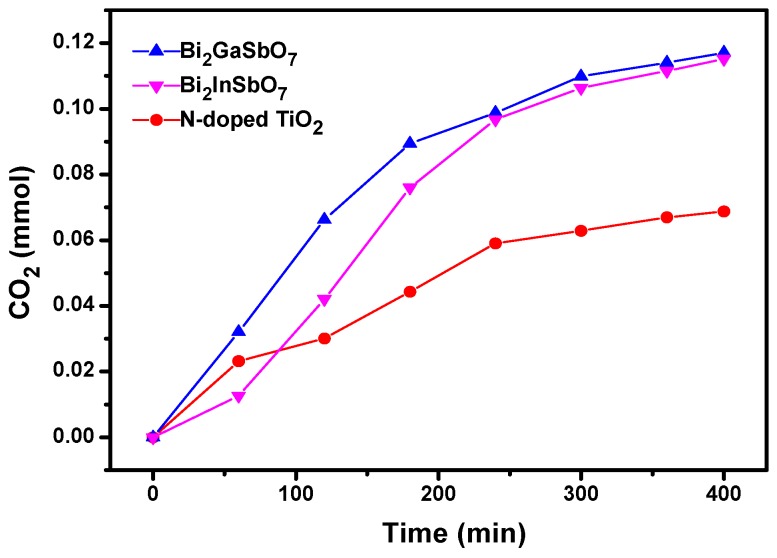
CO_2_ production kinetics during the photocatalytic degradation of methylene blue with Bi_2_GaSbO_7_, Bi_2_InSbO_7_ or N-doped TiO_2_ as a catalyst under visible light irradiation.

Figure 9 shows the amount of variation of CO_2_ produced during the photocatalytic degradation of MB by using Bi_2_GaSbO_7_, Bi_2_InSbO_7_ or N-doped TiO_2_ as a photocatalyst under visible light irradiation. It could be distinctly seen from Figure 9 that the amount of CO_2_ gradually augmented along the light irradiation time and increased less during the last 100 min when much TOC was eliminated according to the results of Figure 8. In addition, after visible light irradiation of 400 min, the CO_2_ production of 0.11711 mmol or 0.11512 mmol with Bi_2_GaSbO_7_ or Bi_2_InSbO_7_ as a catalyst was higher than that of 0.06875 mmol with N-doped TiO_2_ as a catalyst. In addition, the amount of CO_2_ production was nearly equivalent to that of the removed TOC; at the same time, the amount of CO_2_ production or the removed TOC was slightly lower than the amount of reduced MB by using different catalysts with respect to the C element equilibrium, which indicated that MB was mainly degraded into some inorganic products including CO_2_ and eventually H_2_O.

**Figure 10:**
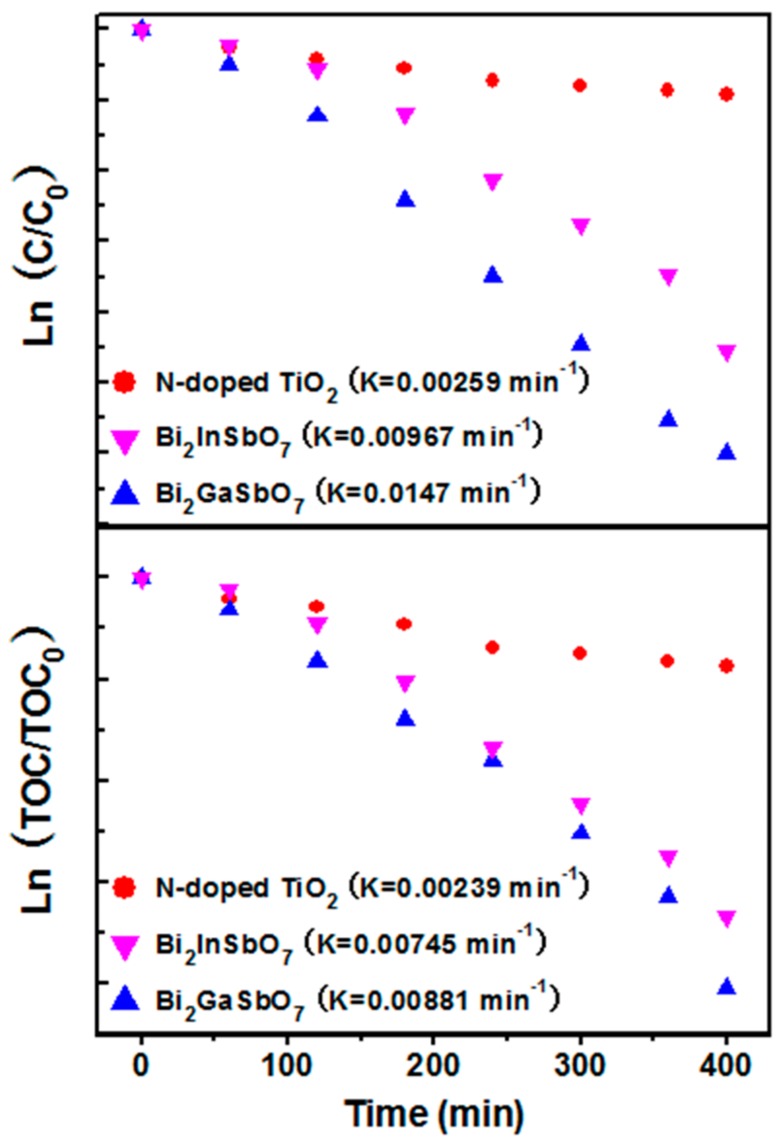
Observed first-order kinetic plots for the photocatalytic degradation of methylene blue with Bi_2_GaSbO_7_, Bi_2_InSbO_7_ or N-doped TiO_2_ as a catalyst under visible light irradiation.

Figure 10 presents the first order nature of the photocatalytic degradation kinetics with Bi_2_GaSbO_7_, Bi_2_InSbO_7_ or N-doped TiO_2_ as a catalyst, which exhibits a linear correlation between ln(*C/C*_0_) or ln(*TOC/TOC*_0_) and the irradiation time for the photocatalytic degradation of MB under visible light irradiation by using the aforementioned catalysts. The pseudo-first-order kinetic curves of MB photodegradation were plotted to quantitatively compare the degradation rate of MB [72]. In the above expression, *C* and *TOC* represented the MB concentration and the total organic carbon concentration at time *t*, respectively. Likewise, *C*_0_ and *TOC*_0_ represented the initial concentration of MB and the initial total organic carbon concentration, respectively. By a linear fit for the relationship between ln(*C/C*_0_) and the irradiation time, the first-order rate constant *k_C_* was estimated to be 0.01470 min^−1^ with Bi_2_GaSbO_7_ as a catalyst, 0.00967 min^−1^ with Bi_2_InSbO_7_ as a catalyst or 0.00259 min^−1^ with N-doped TiO_2_ as a catalyst, which distinctly showed that Bi_2_GaSbO_7_ and Bi_2_InSbO_7_, with the highest and the second highest value of *k_C_*, respectively, exhibited more excellent visible light photocatalytic activities for degrading MB compared with N-doped TiO_2_. Similarly, by a linear fit for the relationship between ln(*TOC/TOC*_0_) and the irradiation time, the first-order rate constant *k_TOC_* was estimated to be 0.00881 min^−1^ with Bi_2_GaSbO_7_ as a catalyst, 0.00745 min^−1^ with Bi_2_InSbO_7_ as a catalyst or 0.00239 min^−1^ with N-doped TiO_2_ as a catalyst. The difference between *k_C_* and *k_TOC_* reflected that there might be some photodegradation intermediate products of MB which were produced during the photocatalytic degradation of MB under visible light irradiation.

**Figure 11:**
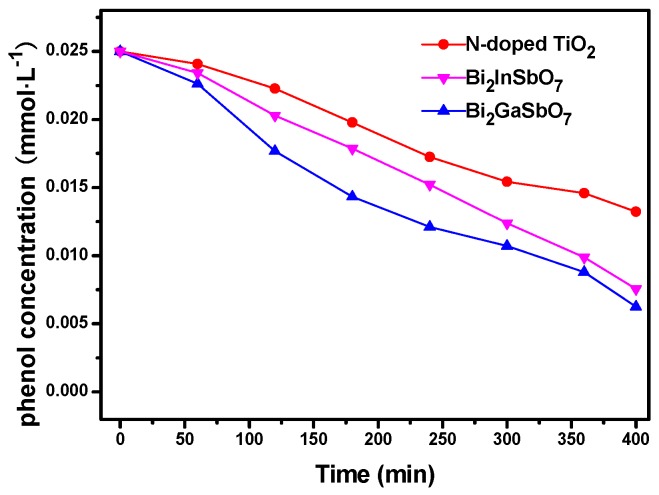
Photocatalytic degradation of phenol under visible light irradiation in the presence of Bi_2_GaSbO_7_, Bi_2_InSbO_7_ or N-doped TiO_2_ as a photocatalyst.

Figure 11 presents the photocatalytic degradation rate of phenol under visible light irradiation in the presence of Bi_2_GaSbO_7_, Bi_2_InSbO_7_ or N-doped TiO_2_ as a photocatalyst with respect to time. It could be seen from Figure 11 that improved activity was obtained when colorless phenol was selected as a contaminant model with Bi_2_GaSbO_7_ or Bi_2_InSbO_7_ as a photocatalyst in comparison with the N-doped TiO_2_. The photocatalytic degradation efficiency of phenol by using Bi_2_GaSbO_7_, Bi_2_InSbO_7_ or N-doped TiO_2_ as a photocatalyst under visible light irradiation after 400 min was estimated to be 75.00%, 69.76% or 47.08%, respectively, indicating that Bi_2_GaSbO_7_ or Bi_2_InSbO_7_ itself had photocatalytic activity and that the photodegradation process of MB by using Bi_2_GaSbO_7_ or Bi_2_InSbO_7_ as a photocatalyst was not mainly due to the photosensitive effect [73]. Moreover, we could observe that the photodegradation efficiency or apparent rate constant of phenol or MB in the presence of Bi_2_GaSbO_7_ or Bi_2_InSbO_7_ was much higher than that in the presence of N-doped TiO_2_, meaning that the visible-light photocatalytic activity of Bi_2_GaSbO_7_ or Bi_2_InSbO_7_ was higher than that of N-doped TiO_2_.

The specific surface area of Bi_2_GaSbO_7_ or Bi_2_InSbO_7_ was measured to be 2.36 m^2^·g^−1^ or 1.82 m^2^·g^−1^, which was much smaller than that of N-doped TiO_2_, whose specific surface area was 45.53 m^2^·g^−1^. Generally speaking, a larger specific surface area would facilitate higher photocatalytic activities at the same experimental condition [74,75]. However, according to preceding results and discussions, Bi_2_GaSbO_7_ and Bi_2_InSbO_7_ showed higher activities than N-doped TiO_2_ for degrading MB during visible light irradiation, which sufficiently highlighted the excellent photocatalytic properties of Bi_2_GaSbO_7_ and Bi_2_InSbO_7_, and the above results might ascribe to two explanations. Firstly, as already mentioned, the calculated band gap for Bi_2_GaSbO_7_, Bi_2_InSbO_7_ or N-doped TiO_2_ was 2.59 eV, 2.54 eV or 2.78 eV. Apparently, Bi_2_GaSbO_7_ or Bi_2_InSbO_7_ possessed a narrower band gap than N-doped TiO_2_, meaning that Bi_2_GaSbO_7_ or Bi_2_InSbO_7_ could utilize more visible light energy than N-doped TiO_2_ [76,77]. Secondly, according to the XRD results of Bi_2_GaSbO_7_ and Bi_2_InSbO_7_, we could find that Bi_2_GaSbO_7_ and Bi_2_InSbO_7_ were both obtained with high crystallization perfection, which might more efficiently inhibit the recombination of photoinduced electrons and holes than N-doped TiO_2_.

Meanwhile, the photocatalytic degradation rate and photonic efficiency of Bi_2_GaSbO_7_ were slightly higher than that of Bi_2_InSbO_7_. There were perhaps two probable reasons to explain it. As we all know, the greater mobility of the photoinduced electrons and holes indicated the greater chance that the photoinduced electrons and holes would reach the reactive sites of the catalyst surface, which would bring higher photocatalytic activities. As we previously mentioned, the lattice parameter *α* = 10.356497 Å for Bi_2_GaSbO_7_ was lower than the lattice parameter *α* = 10.666031 Å for Bi_2_InSbO_7_. Generally speaking, the smaller the ionic radius was, the smaller the size of the particles could be; and the lower the lattice parameter was, the larger the specific surface area could be, which could increase more reactive sites on the photocatalyst surface and absorb more reactive species to improve the photocatalytic activities [78]. In addition, according to previous luminescent studies, the closer the M–O–M bond angle was to 180°, the more delocalized the excited state was [79]. As a result, the charge carriers could move easily in the matrix. In this experiment, for Bi_2_GaSbO_7_, the Ga–O–Ga bond angle was 131.302°; accordingly, for Bi_2_InSbO_7_, the In–O–In bond angle was 128.640°. Obviously, the bond angle of the Ga–O–Ga bond angle of Bi_2_GaSbO_7_ was larger than the bond angle of Bi_2_InSbO_7_, which induced that Bi_2_GaSbO_7_ exhibited higher photocatalytic activity than Bi_2_InSbO_7_.

### 3.3. Photocatalytic Degradation Pathway of MB with Bi_2_GaSbO_7_ and Bi_2_InSbO_7_ as Photocatalysts

**Figure 12:**
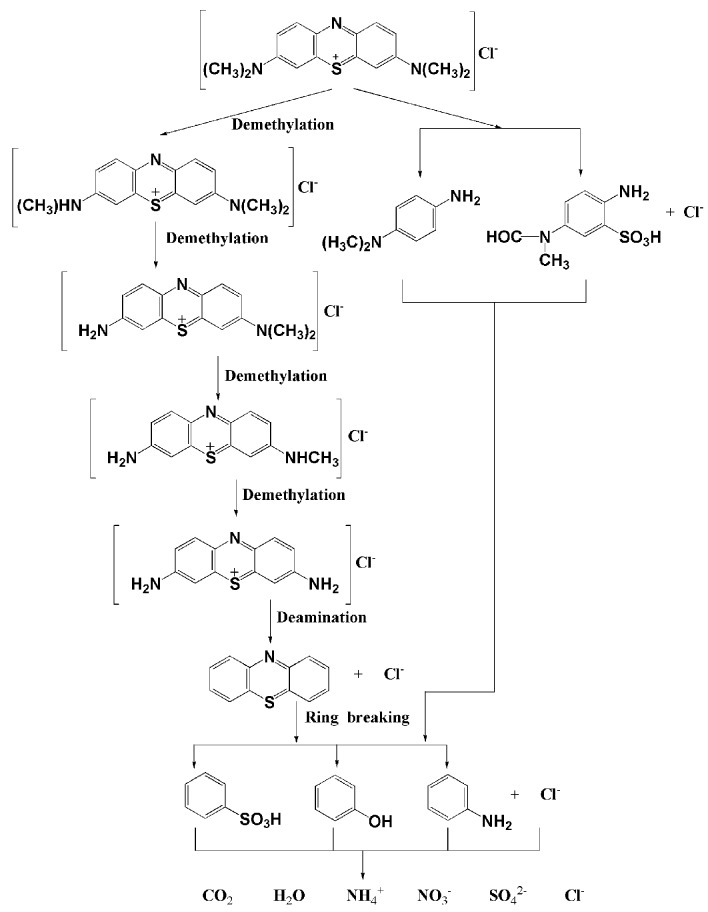
Suggested photocatalytic degradation pathway scheme for methylene blue under visible light irradiation in the presence of Bi_2_GaSbO_7_ or Bi_2_InSbO_7_.

The photodegradation intermediate products of MB in our experiment were identified as azure A, azure C, thionine, phenothiazine, leucomethylene blue, *N*,*N*-dimethyl-*p*-phenylenediamine, benzenesulfonic acid, phenol and aniline. There generated holes h^+^, **·**O_2_^−^ and ·OH radicals, as oxidative agents in the photocatalytic reactions. According to previous studies [80,81], the photodegradation of MB might occur by demethylation. Besides, there were also reports [82] which pointed out that ·OH radicals would first attack C − S^+^ = C functional group bonds to open the central aromatic ring which contained both heteroatoms S and N. Therefore, according to previous studies and our test results, a possible photocatalytic degradation pathway for MB was proposed. Figure 12 shows the suggested photocatalytic degradation pathway scheme for MB under visible light irradiation with Bi_2_GaSbO_7_ or Bi_2_InSbO_7_ as a catalyst. The MB molecule was converted to small organic species, which were subsequently mineralized into inorganic products such as SO_4_^2−^ ions, NO_3_^−^ ions, CO_2_ and ultimately water.

### 3.4. Photocatalytic Degradation Mechanism

**Scheme 1:**
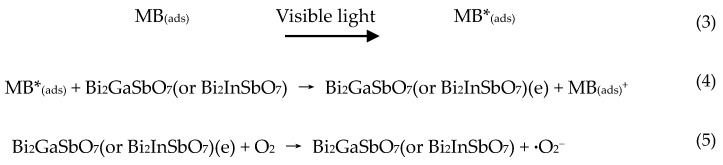
The photosensitization effect by the dye MB.

**Figure 13:**
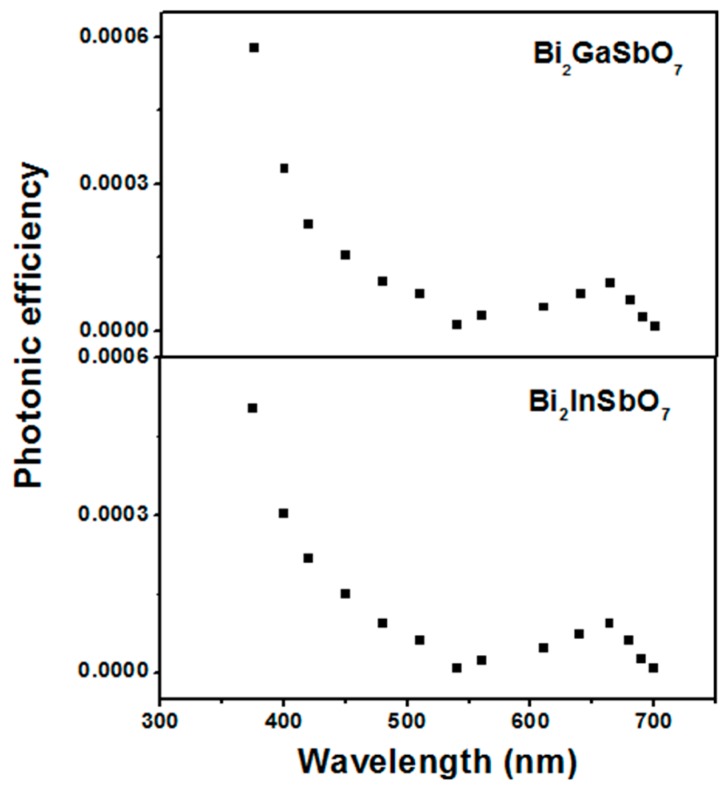
Action spectra of methylene blue degradation with Bi_2_GaSbO_7_ or Bi_2_InSbO_7_ as a catalyst under visible light irradiation.

Figure 13 presents the action spectra of MB degradation with Bi_2_GaSbO_7_ or Bi_2_InSbO_7_ as a catalyst under visible light irradiation. A clear photonic efficiency (0.00964% for Bi_2_GaSbO_7_ and 0.00942% for Bi_2_InSbO_7_ at their respective maximal point) at wavelengths which corresponded to sub-*E*_g_ energies of the photocatalysts (*λ* from 480 to 700 nm for Bi_2_GaSbO_7_ and *λ* from 490 to 700 nm for Bi_2_InSbO_7_) was observed. The existence of photonic efficiency at this region revealed that the photons were not absorbed by the photocatalysts. Enlightened by the correlation between the low-energy action spectrum and the absorption spectrum of MB, we speculated that any photodegradation which results at wavelengths above 480 nm, should be attributed to photosensitization effect by the dye MB itself (Scheme 1). According to the photosensitization scheme, MB which was adsorbed on Bi_2_GaSbO_7_ or Bi_2_InSbO_7_ was excited by visible light irradiation. Subsequently, an electron was injected from the excited MB to the conduction band of Bi_2_GaSbO_7_ or Bi_2_InSbO_7_ where the electron was scavenged by molecular oxygen. This explained the results which were gained with Bi_2_GaSbO_7_ or Bi_2_InSbO_7_ as a catalyst under visible light irradiation, where the catalyst could serve to reduce recombination of photoinduced electrons and photoinduced holes by scavenging of electrons.

**Scheme 2:**
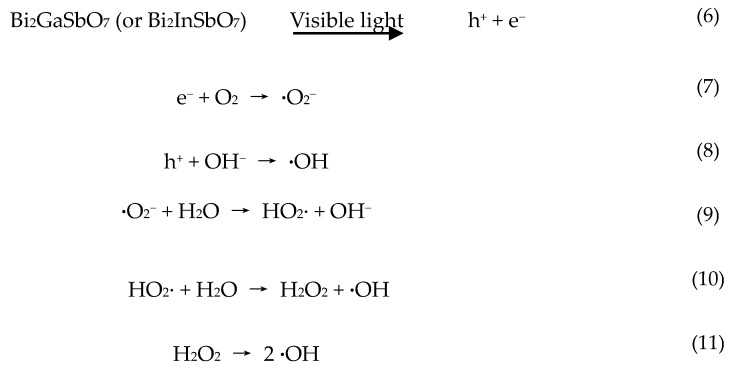
The production scheme of oxidative radicals with Bi_2_GaSbO_7_ or Bi_2_InSbO_7_ as catalyst.

The situation was different below 480 nm, where the photonic efficiency correlated well with the absorption spectra of Bi_2_GaSbO_7_ or Bi_2_InSbO_7_. This result evidently indicated that the mechanism was the photodegradation of MB by the band gap excitation of Bi_2_GaSbO_7_ or Bi_2_InSbO_7_. As already mentioned, holes of h^+^, **·**O_2_^−^ and OH· radicals served as oxidative agents in the photocatalytic reactions. Although the detailed experiments about the effect of oxygen and water on the degradation mechanism of MB were not performed, it was sensible to assume that the mechanism in the first step was similar to the observed mechanism for Bi_2_GaSbO_7_ or Bi_2_InSbO_7_ under supra-bandgap irradiation, and the production scheme of oxidative radicals commonly was shown below (Scheme 2).

**Figure 14:**
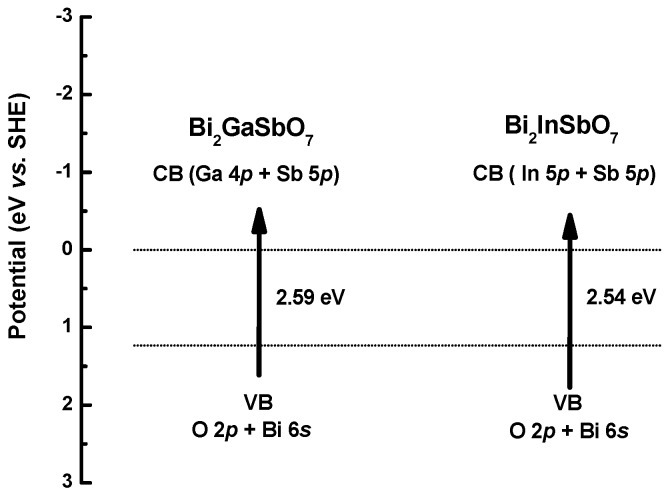
Suggested band structures of Bi_2_GaSbO_7_ and Bi_2_InSbO_7_.

Figure 14 shows the suggested band structures of Bi_2_GaSbO_7_ and Bi_2_InSbO_7_. The positions and width of the conduction band (CB) and the valence band (VB) were studied by calculating the electronic band structure of Bi_2_GaSbO_7_ or Bi_2_InSbO_7_ with the plane-wave-based density functional method. The band structure calculations of Bi_2_GaSbO_7_ and Bi_2_InSbO_7_ were carried out with the program of Cambridge serial total energy package (CASTEP) and first-principles simulation. It could be seen from Figure 14 that the conduction band of Bi_2_GaSbO_7_ was composed of Ga 4*p* and Sb 5*p* orbital component, meanwhile, the valence band of Bi_2_GaSbO_7_ was composed of a small dominant O 2*p* and Bi 6*s* orbital component. Similarly, the conduction band of Bi_2_InSbO_7_ was composed of In 5*p* and Sb 5*p* orbital component. In addition, the valence band of Bi_2_InSbO_7_ was composed of a small dominant O 2*p* and Bi 6*s* orbital component. Direct absorption of photons by Bi_2_GaSbO_7_ or Bi_2_InSbO_7_ could produce electron–hole pairs within the catalyst, indicating that the larger energy than the band gap of Bi_2_GaSbO_7_ or Bi_2_InSbO_7_ was necessary for decomposing MB by the photocatalysis method. 

## 4. Conclusions 

New photocatalysts Bi_2_GaSbO_7_ and Bi_2_InSbO_7_ were firstly prepared by the solid-state reaction method. The structural properties and optical absorption properties of Bi_2_GaSbO_7_ and Bi_2_InSbO_7_ were characterized by some material characterization methods, the photocatalytic properties of Bi_2_GaSbO_7_ and Bi_2_InSbO_7_ were also verified in comparison with N-doped TiO_2_. XRD results indicated that Bi_2_GaSbO_7_ and Bi_2_InSbO_7_ crystallized with the pyrochlore-type structure, cubic crystal system and space group *Fd3m*. The lattice parameter *a* for Bi_2_GaSbO_7_ or Bi_2_InSbO_7_ was *a* = 10.356497 Å or *a* = 10.666031 Å. According to the results from the UV-vis absorption spectra of Bi_2_GaSbO_7_ and Bi_2_InSbO_7_, the band gap of Bi_2_GaSbO_7_ or Bi_2_InSbO_7_ was estimated to be about 2.59 eV or 2.54 eV, indicating that Bi_2_GaSbO_7_ and Bi_2_InSbO_7_ showed a strong optical absorption in the visible light region (*λ* > 420 nm). Photocatalytic degradation of aqueous MB was realized under visible light irradiation in the presence of Bi_2_GaSbO_7_ or Bi_2_InSbO_7_ accompanied with the formation of final products such as CO_2_ and water. The complete removal of organic carbon from MB was obtained as indicated from TOC and CO_2_ yield measurements with Bi_2_GaSbO_7_ or Bi_2_InSbO_7_ as a catalyst under visible light irradiation. Compared with N-doped TiO_2_, Bi_2_GaSbO_7_ and Bi_2_InSbO_7_ exhibited higher photocatalytic activities for MB degradation under visible light irradiation. Consequently, according to the above analyses, Bi_2_GaSbO_7_ and Bi_2_InSbO_7_ both had great potential to degrade MB in textile industry wastewater. In addition, Bi_2_GaSbO_7_ exhibited slightly higher photocatalytic activities for the degradation of MB than Bi_2_InSbO_7_.

## Figures and Tables

**Table 1:** Binding energies (BE) for key elements of Bi_2_InSbO_7_ and Bi_2_GaSbO_7_.

Compound	Bi_4f7/2_ BE (eV)	Sb_3d5/2_ BE (eV)	Ga_3d5/2_ BE (eV)	In_3d5/2_ BE (eV)	O_1s_ BE (eV)
Bi_2_InSbO_7_	159.70	531.20	–	444.60	530.85
Bi_2_GaSbO_7_	159.60	531.40	20.60	–	531.10

**Table 2:** Structural parameters of Bi_2_GaSbO_7_ prepared by the solid state reaction method.

Atom	x	y	z	Occupation Factor
Bi	0.00000	0.00000	0.00000	1.0
Ga	0.50000	0.50000	0.50000	0.5
Sb	0.50000	0.50000	0.50000	0.5
O(1)	−0.18500	0.12500	0.12500	1.0
O(2)	0.12500	0.12500	0.12500	1.0

**Table 3:** Structural parameters of Bi_2_InSbO_7_ prepared by the solid state reaction method.

Atom	x	y	z	Occupation Factor
Bi	0.00000	0.00000	0.00000	1.0
In	0.50000	0.50000	0.50000	0.5
Sb	0.50000	0.50000	0.50000	0.5
O(1)	−0.16500	0.12500	0.12500	1.0
O(2)	0.12500	0.12500	0.12500	1.0
